# The Impact of Body Composition on Mortality and Hospital Length of Stay after Endovascular and Open Aortic Aneurysm Repair: A Retrospective Cohort Study

**DOI:** 10.3390/nu16183205

**Published:** 2024-09-22

**Authors:** Rosaria Del Giorno, Alessandro Robaldo, Alessia Astorino, Luca Gabutti, Vito Chianca, Stefania Rizzo, Francesca Riva, Ludovica Ettorre, Kevyn Stefanelli, Reto Canevascini, Luca Giovannacci, Giorgio Prouse

**Affiliations:** 1 Faculty of Biomedical Science, Università Della Svizzera Italiana, USI-Lugano, 6900 Lugano, Switzerland; alessia.astorino@hotmail.com (A.A.); luca.gabutti@eoc.ch (L.G.); stefania.rizzo@eoc.ch (S.R.); 2 Department of Vascular Surgery and Angiology, Ente Ospedaliero Cantonale, 6900 Lugano, Switzerland; alessandro.robaldo@eoc.ch (A.R.); francesca.riva@eoc.ch (F.R.); ludovica.ettorre@eoc.ch (L.E.); reto.canevascini@eoc.ch (R.C.); luca.giovannacci@eoc.ch (L.G.); giorgio.prouse@eoc.ch (G.P.); 3 Family Medicine Institute, University of Southern Switzerland, 6900 Lugano, Switzerland; vito.chianca@eoc.ch; 4 Imaging Institute of Italian Switzerland, Ente Ospedaliero Cantonale, 6900 Lugano, Switzerland; 5 Department of Social Sciences and Economics, Sapienza University of Rome, 00185 Rome, Italy; kevyn.stefanelli@gmail.com

**Keywords:** sarcopenia, abdominal aortic aneurysm, endovascular aneurysm repair, aortic open surgical repair

## Abstract

**Background:** Sarcopenia is an indicator of preoperative frailty and a patient-specific risk factor for poor prognosis in elderly surgical patients. Some studies have explored the prognostic significance of body composition parameters in relation to perioperative mortality after aortic repair and to mid- and long-term survival following endovascular aneurysm repair (EVAR). This study aimed to comprehensively investigate the effects of various body composition parameters, including but not limited to sarcopenia, on short- and long-term mortality as well as the length of hospital stay in two large cohorts of patients undergoing open surgical aortic repair (OSR) or EVAR. **Methods:** A single-institution retrospective cohort study included patients who underwent EVAR or OSR from January 2010 to December 2017. Several parameters of body composition on axial CT angiography images were analyzed, such as skeletal muscle area (SMA) with derived skeletal muscle index (SMI), visceral adipose tissue (VAT), and subcutaneous adipose tissue (SAT). **Results:** 477 patients were included: 250 treated by OSR and 227 by EVAR; the mean age was 70.8 years (OSR) and 76.3 years (EVAR), with a mean follow-up of 54 months. Sarcopenia was associated with a prolonged length of hospital stay in EVAR patients but not in OSR patients (β coefficient 3.22; *p*-value 0.022 vs. β coefficient 0.391; *p*-value 0.696). Sarcopenia was an elevated one-year mortality risk post-EVAR compared to those without sarcopenia (*p*-value for the log-rank test 0.05). SMA and SMI were associated with long-term mortality in EVAR patients even after adjusting for multiple confounders (HR 0.98, *p*-value 0.003; HR 0.97, *p*-value 0.032). The analysis of the OSR cohort did not show a significant correlation between short- and long-term mortality and sarcopenia indicators. **Conclusions:** The results suggest that body composition could predict increased mortality and longer hospital stays in patients undergoing EVAR procedures. These findings were not confirmed in the cohort of patients who underwent OSR. Patients with sarcopenia and pre-operative malnutrition should be critically assessed to define the indication for treatment in this predominantly elderly and morbid cohort, despite EVAR procedures being less invasive. Body composition evaluation is an inexpensive and reproducible tool that can contribute to an improved decision-making process by identifying patients who will benefit most from EVAR, ensuring a more personalized and cost-effective treatment strategy. Further studies are planned to explore the added value of integrating body composition into a comprehensive risk stratification before aortic surgery.

## 1. Background

Abdominal aortic aneurysm (AAA) is a potentially life-threatening condition due to the risk of rupture [1,2]. Endovascular aneurysm repair (EVAR) and open surgical repair (OSR) are the primary treatments for aortic aneurysms with a maximum diameter ≥ 50 mm in women and ≥ 55 mm in men [3].

Both techniques are well-established and allow for a tailored, patient-centered approach to treating a wide range of patients with aortic aneurysms. However, there is still a lack of solid evidence on the survival advantage for patients selected for repair solely based on aneurysm size [3]. This suggests that there may be a subgroup of patients, indicated for treatment based on aneurysm size, who might benefit more from conservative treatment. 

Currently, few risk stratification models accurately estimate late survival after EVAR and OSR, often lacking significant prognostic factors [4,5].

Sarcopenia, or low muscle mass, has emerged as a crucial factor in preoperative decision-making for surgical eligibility across various specialties [6,7].

Evidence also suggests its role as an independent risk factor for decreased long-term survival after EVAR [8]. Preoperative CT studies measuring sarcopenia could predict adverse operative outcomes and aid in selecting patients who will benefit most from surgical treatment [9,10].

Several studies have found a significant association between low skeletal muscle mass and mortality in patients after EVAR. However, other studies analyzing the correlation of body composition with survival after aortic repair, including both EVAR and OSR, did not find a significant correlation [11,12].

Conflicting evidence exists regarding measurement methods and populations [9,10]. While computed tomography (CT) scans are universally utilized for preoperative assessment in AAA patients [13], previous studies have predominantly focused on the relationship between the psoas muscle and outcomes after aortic repair, neglecting the influence of other muscle groups such as those in the anterior and lateral abdomen [10].

Despite extensive exploration of the prognostic role of low muscle mass in outcomes after EVAR, there remains a scarcity of research investigating the prognostic significance of body composition parameters after OSR. In most centers, the cohort of patients treated by OSR differs significantly in terms of age and comorbidities from patients treated by EVAR, making results from EVAR studies not applicable to OSR patients.

In this study, we aimed to fill this gap by analyzing body composition in preoperative CT scans, including subcutaneous fat, visceral fat, lean muscle, and intramuscular fat. The study aimed to investigate the prognostic significance of various body composition parameters on medium- and long-term survival, as well as the length of hospital stay, by analyzing patients undergoing EVAR or OSR separately. Through this comprehensive analysis, we sought to provide valuable insights that can inform clinical decision-making in the management of AAAs.

## 2. Methods

### 2.1. Study Patients

A retrospective cohort study was conducted on all patients who underwent elective EVAR or OSR for AAA at a teaching hospital in Switzerland between January 2010 and December 2017. All procedures adhered to current guidelines for determining optimal treatment for infrarenal AAA. The surgical criteria for AAA repair were based on international guidelines [14] and defined as follows: a maximum diameter ≥ 55 mm in men and ≥50 mm in women; an enlargement of ≥10 mm within 12 months; unruptured symptomatic AAA; and saccular aneurysms with a maximum diameter < 55 mm if deemed at increased risk of rupture by multidisciplinary evaluation.

The selection criteria for EVAR or OSR procedures at our department were based on several parameters, including anatomical criteria, age (elderly patients over 75 years of age vs. younger patients), presence or absence of significant comorbidities, surgical risk, and history of major abdominal surgery.

This study was conducted in accordance with the mandates of the Declaration of Helsinki. Approval for the study was obtained from the Local Swiss Ethics Committee (approval number 2022-01553-4164, approved on 27 October 2023).

### 2.2. Data Collection

Demographic and clinical information were extracted from electronic medical records. Clinical information was independently reviewed by two trained reviewers directly from the electronic medical records. Comorbidities collected included cancer, diabetes mellitus, hypertension, coronary artery disease, chronic obstructive pulmonary disease, stroke or transient ischemic attack (TIA), congestive heart failure, chronic kidney disease, obesity (based on body mass index), and hyperlipidemia.

Coronary artery disease was defined by the presence of one or more of the following: history of myocardial infarction; prior coronary bypass or percutaneous coronary intervention; a 12-lead electrocardiography showing evidence of ischemia. Congestive heart failure was determined based on a history of ejection fraction < 50% on echocardiography. Stroke or TIA was categorized as a documented diagnosis, history of TIA, or history of cerebrovascular accident. Hyperlipidemia was assessed through a prior diagnosis of elevated lipids or the prescription of statins at the time of surgery.

Finally, mortality data were collected after the completion of all computed tomography (CT) imaging analysis to ensure blindness to outcomes during radiologic analysis.

Blinding was implemented in order to attenuate potential subjective biases in image interpretation by ensuring that CT analysis was carried out exclusively on the basis of imaging data, regardless of knowledge of patient outcomes.

### 2.3. Definitions

Preoperative contrast-enhanced CT were performed within 3 months from EVAR or OSR and available in digital format in medical records. CT scan analysis was used for body composition measurements. 

A trained radiologist selected an axial CT image at the level of the third lumbar vertebra (L3), which was stored in digital format and then uploaded into the Slice-O-Matic software v 5.0 (Tomovision, Montreal, QC, Canada). An additional automatic module for segmentations (ABACS, Tomovision, Montreal, QC, Canada) was firstly used. In the few cases where the automatic segmentation was not considered appropriate, the segmentation was manually adjusted by the radiologist. CT attenuation thresholds for the automatic module were −29 to 150 Hounsfield Units (HU) for skeletal muscle; −190 to −30 HU for subcutaneous adipose tissue; and −150 to −50 HU for visceral adipose tissue. The following quantitative measures were recorded: skeletal muscle area (SMA) as area (cm^2^), skeletal muscle density (SMD) as HU, subcutaneous adipose tissue (SAT) as area (cm^2^), and visceral adipose tissue (VAT) as area (cm^2^). Skeletal muscle index (SMI) was calculated by normalizing SMA by square height (m^2^) and reported as cm^2^/m^2^. The muscle area included the psoas, erector spinae, quadratus lumborum, transversus abdominis, external obliques, internal obliques, and rectus abdominis muscles. 

Sarcopenia was defined as a skeletal muscle area < 114.0 cm^2^, based on a definition previously established in the literature [15,16,17]. This threshold corresponds to the fifth percentile of standard muscle mass measurements in healthy adults [18]. It was initially derived from donor liver transplantation data and later applied to assess sarcopenia in patients with various medical conditions [15,16,17].

It is, however, important to note that sarcopenia remains a poorly defined entity. To further investigate its impact, we analyzed muscle mass in EVAR and OSR patients, considering total skeletal muscle area as a continuous variable.

### 2.4. Statistical Analysis

Descriptive statistics are presented as mean ± SD for continuous variables and as number (percentage) for categorical variables. Differences among EVAR and OSR procedures were analyzed by analysis of variance and χ^2^ tests for continuous and categorical variables, respectively.

Sarcopenia was defined as having a skeletal muscle area <114.0 cm^2^. This definition was originally derived from data in donor liver transplantation patients and later used to assess sarcopenia in CLI patients and to explore the impact of sarcopenia following endovascular aneurysm repair [14,15,16].

To investigate the association between length of hospital stay and body composition parameters in EVAR and OSR, linear regression models were constructed. The β coefficients and confidence intervals were calculated.

The 1-year and long-term survival (i.e., freedom from all-cause mortality) rate was estimated using Kaplan–Meier curves. Differences in Kaplan–Meier survival curves were analyzed using a log-rank test. Univariate and multivariate Cox proportional hazards models were used to examine mortality for unadjusted and adjusted associations with body composition parameters at 1-year and long-term. Resulting Hazard ratios and 95% confidence intervals were calculated for each covariate in the multivariate analysis. Crude (Model 1) and adjusted (Model 2) were created for both linear and cox regression models. Model 2 was adjusted for selected covariates to examine potential confounding effects. Data analysis was performed using All statistical analysis was done in R version 3.4.3 (RCoreTeam, 2017) and SPSS (version 18.0, Chicago, IL, USA). Statistical significance for all outcomes was set at *p* ≤ 0.05.

## 3. Results

### 3.1. Characteristics of the Patients

A total of 477 patients underwent EVAR or OSR for an AAA at our institution between January 2010 and December 2017. A total of 227 patients (47.6%) underwent EVAR and 250 (52.4%) OSR; 431 (90.4%) were male without differences between procedures (EVAR vs. OSR) in sex distribution (*p*-value 0.308), overall mean age 73.4 ± 8.0 years, and 76.3 ± 7.5 in the EVAR group vs. 70.8 ± 7.6 in OSR cohort (*p*-value < 0.001).

Comorbidities and baseline characteristics of the study population, with differences between EVAR and OR groups, are shown in Table 1.

The mean muscle area (SMA) for all patients was 146.6 ±33.5 cm^2^, without significant differences between procedure cohorts (EVAR vs. OSR): 143.5 ± 31.2 cm^2^ vs. 149.5 ± 35.1 cm^2^; *p*-value 0.085. Similarly, non-significant differences between the two cohorts were found for other body composition parameters as well. Regarding genders, 46 patients (9.6%) out of the total population were females. The gender distribution of body composition parameters are illustrated in Supplementary Table S1.

Sarcopenic patients were significantly older, with a reduced BMI and with a lower diabetes prevalence. Differences among sarcopenic vs. non-sarcopenic patients are depicted in Supplementary Table S3.

### 3.2. Length of Hospital Stay

The association between length of hospital stay (LOS) and body composition parameters was investigated using multivariate linear regression models. Table 2 shows the linear associations between all body composition parameters and LOS. A sub-analysis by gender was also performed and is illustrated in Supplementary Table S2.

Patients treated with EVAR showed a significant association between LOS and SMA and sarcopenia, in both the crude model and the adjusted model, respectively: β = −0.200 (95% CI, −0.111; −0.017), *p* < 0.001; β = −0.194 (95% CI −0.114; −0.010), *p* = 0.019; and β = −0.247 (95% CI, −2.853; −11.013), *p* < 0.001, and −3.224 (−2.734–11.370) *p* = 0.002.

### 3.3. Overall and 1-Year Mortality

Throughout the entire follow-up period, a total of 225 deaths were observed: 103 deaths (41% of the cohort) in the OSR group and 122 deaths (53% of the cohort) in the EVAR group. After one year of follow-up, 42 deaths were observed, representing 9% of the study population. Of these, 33 deaths (15%) occurred in the OSR treatment group, and 9 deaths were in the EVAR treatment group. After five years, the total number of deaths increased to 127, or 27% of the total population: 60 deaths (24%) were in the OSR group and 67 deaths (29%) were in the EVAR group (*p*-value 0.023).

**Figure 1:**
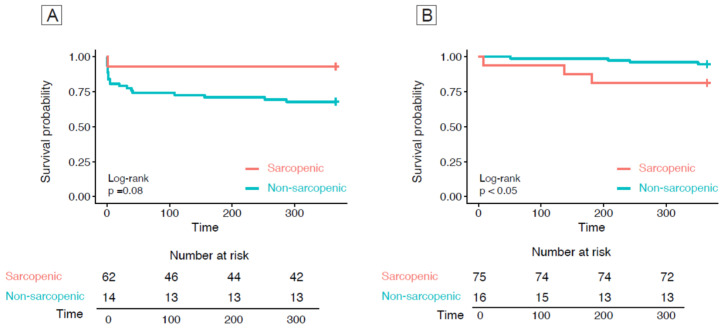
Kaplan–Meier curves for 1-year survivals in OSR (**A**) and in EVAR (**B**) according to sarcopenic/non-sarcopenic status.

The Kaplan–Meier estimate for 1-year mortality in EVAR and OSR according to the status of sarcopenia/not sarcopenia showed a significant difference in survival probability in sarcopenic vs. non-sarcopenic patients in EVAR (Figure 1B, *p*-value 0.005). In the OSR cohort, differences according to sarcopenic status were non-significant (Figure 1A; *p*-value 0.08). 

**Figure 2:**
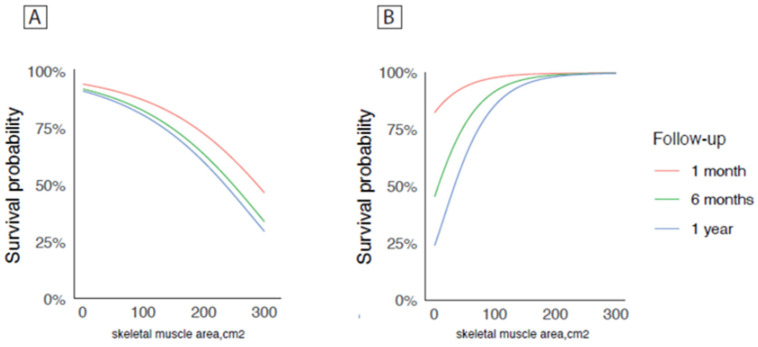
Kaplan–Meier estimate for 1-month, 6-month, and 1-year mortality in EVAR and OSR considering values of SMA as a continuous variable. A significant difference in survival probability according to SMA values (*p*-values < 0.001) was found in OSR (**A**) and in EVAR (**B**).

Kaplan–Meier estimate for 1-month, 6-month, and 1-year mortality in EVAR and OSR was explored also considering the values of SMA as a continuous variable (Figure 2). Figure 2A (OSR) and Figure 2B (EVAR) show a significant difference in survival probability according to SMA values (*p*-values < 0.001).

Multivariable Cox regression analysis of 1-year mortality confirmed a significant association between body composition and 1-year mortality in EVAR patients. Specifically, SMA was associated with a significant increase in risk of mortality at 1 year in EVAR patients (HR 1.31; *p*-value 0.026) (Table 3).

The multivariable Cox regression analysis of long-term survival demonstrated that body composition parameters were associated with long-term mortality in EVAR patients. With increasing skeletal muscle area, skeletal muscle size, and visceral adipose tissue, a significantly decreased risk of long-term mortality was found. Notably, SMA was significantly associated with long-term mortality in both the crude and adjusted models, showing an HR of 0.99 in both models (*p*-value 0.004: 0.003, respectively).

Furthermore, SMI and VAT were also significantly associated with long-term mortality with HRs of 0.98 and 0.99, respectively (*p*-value of 0.032 and 0.034, respectively) (Table 4).

**Figure 3:**
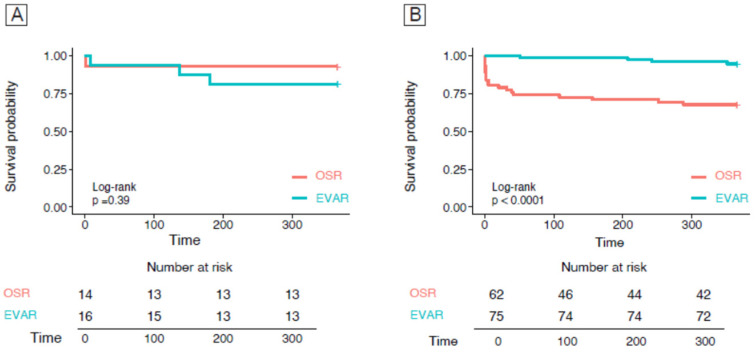
Kaplan–Meier curves for 1-year survivals in sarcopenic (**A**) and non-sarcopenic patients (**B**) in the EVAR and OSR groups.

The estimated 1-year mortality for OSR and EVAR according to the presence or absence of sarcopenia is represented in Figure 3. Non-sarcopenic patients treated with EVAR showed significantly better survival compared to OSR, mostly due to the increased peri-operative mortality of OSR (Figure 3B, *p*-value < 0.001).

## 4. Discussion

The present study identifies body composition and sarcopenia as independent predictors of intermediate-term and long-term mortality in patients undergoing EVAR for AAA. To the best of our knowledge, this study is the most comprehensive to date, examining various body composition parameters and their impact on outcomes after AAA repair.

It uniquely explores these associations separately in both pivotal procedures for aortic repair: OSR and EVAR. Additionally, this study offers novel insights showing the prognostic value of body composition parameters and sarcopenia in predicting the length of hospital stay in patients treated with EVAR.

The novelty of this research is highlighted by its analysis across two distinct aortic repair techniques, allowing for a more nuanced understanding of how body composition affects surgical outcomes in both contexts. Unlike previous studies that have often focused on isolated aspects of body composition or limited their scope to a single procedure, this study integrates a wide range of metrics from preoperative CT scans and assesses their effects on both short- and long-term outcomes. This comprehensive approach not only advances our understanding of the role of sarcopenia and other body composition parameters but also emphasizes the need for tailored treatment strategies based on detailed preoperative assessments.

Improving prognostic accuracy in patients undergoing AAA repair is a crucial and clinically relevant goal to optimize healthcare services. Decisions about conservative versus surgical treatments of AAAs are currently based on aneurysm size. The growing evidence that AAAs with a maximum diameter < 55 mm should be treated conservatively does not provide evidence that the surgical treatment of aneurysms ≥ 55 mm is beneficial. Guidelines have recently downgraded the recommendation for the surgical treatment of aneurysms ≥ 55 mm from level I to level IIa with evidence level C [3].

A more accurate stratification of patients with AAAs ≥ 55 mm could help identify those who may benefit more from conservative treatment than from surgery.

Sarcopenia has emerged as a significant indicator of 30-day mortality and reduced long-term survival among patients undergoing EVAR and complex EVAR procedures for AAAs [17,18,19,20].

Some studies have revealed a 5-year mortality rate nearing 50% following elective EVAR among sarcopenic patients [21,22].

The present study demonstrates a 1-year mortality rate >50% among sarcopenic patients treated with EVAR, supporting and strengthening the existing evidence. This finding was not confirmed in patients treated with OSR. Although several studies have analyzed the risk of sarcopenia in EVAR patients, only a few have reported mid- and long-term results for patients treated by OSR. Interestingly, two studies that analyzed EVAR and OSR patients in the same cohort did not find a significant correlation of sarcopenia with mortality [11,12].

Together with the results on OSR patients in our study, this may suggest that sarcopenia becomes relevant in a more elderly and morbid cohort of surgical patients with AAA, who are typically treated by EVAR.

Multiple characteristics of patients have been suggested to contribute to 1-year mortality after EVAR. Sarcopenia offers a tool to stratify patients’ risk using objective measures obtained from CT images. It offers promising potential either as a stand-alone metric or combined with other clinical and demographic patient characteristics. This integration could improve the accuracy and usefulness of risk assessment in clinical practice to decide which patients to treat with EVAR and which are better served with the best medical treatment strategy.

While sarcopenia may be a valuable tool for patient assessment, the underlying causes of decreased skeletal muscle mass and strategies for its improvement remain lightly explored. Although it seems reasonable that increasing muscle mass and targeting sarcopenia prior to surgery may improve outcomes, there currently is a lack of supporting data [23].

Gender differences also significantly influence both body composition and the prevalence of AAAs, subsequently affecting surgical outcomes. Epidemiological studies indicate that men are disproportionately affected by AAAs, with prevalence rates estimated to be 4–5 times higher compared to women. This gender disparity is thought to arise from a combination of risk factors, including smoking, hypertension, and genetic predispositions [24,25]. In addition, body composition varies markedly between men and women. Women, particularly post-menopausal women, tend to exhibit higher body fat percentages and lower muscle mass relative to men [26]. These differences have important clinical implications, as traditional indices, such as body mass index, may fail to accurately capture variations in muscle mass or fat distribution between genders. Post-menopausal women, for instance, are more susceptible to sarcopenic obesity—a condition characterized by diminished skeletal muscle mass coupled with elevated fat mass. This phenotype has been linked to unfavorable surgical outcomes, particularly in the context of complex interventions such as AAA repair [27,28,29,30].

Although this study confirms more unfavorable outcomes following elective EVAR in patients with sarcopenia, further research is essential to explore methods to improve preoperative skeletal muscle mass and to ascertain whether interventions such as “preventive rehabilitation” can influence outcomes in these patients.

This study has some limitations. There is a lack of consensus on which threshold should be chosen to categorize sarcopenia. We based our choice on the existing literature, but previous studies have not agreed upon the method to determine the thresholds. Moreover, the retrospective nature of data collection relied on the accuracy and completeness of electronic medical records, potentially introducing bias. Furthermore, the segmentation of the body composition measurements on CT images was mainly performed using an automatic tool, which may sometimes lack precision, in favor of reproducibility. However, to overcome this limitation, a radiologist always double-checked the automatic segmentation and corrected it with a semi-automatic tool if necessary. Despite efforts to detect various comorbidities, residual confounding factors may persist, including differences in drug regimens that were not analyzed in this study. Another key limitation of this study is the relatively short follow-up period, which poses challenges common to all long-term medical analyses. Over time, the effects of treatments may be influenced by external variables, potentially masking the true differences highlighted by our statistical findings. Furthermore, the relatively small sample size limits the generalizability of the results. To address these limitations, future research will focus on expanding both the sample size and the duration of follow-up, aiming to achieve more robust and reliable results, particularly for medium- and long-term outcomes.

Finally, the small sample size of women significantly affected our ability to stratify and analyze the results by gender. This limitation aligns with the known disparity in the prevalence of AAAs between genders, as women are less frequently affected than men.

## 5. Conclusions

The results of this study highlight the promising potential of CT-based body composition analysis as a prognostic indicator for patients with AAA who are considered for treatment based on aneurysm size. Body composition assessment could offer a relatively simple and inexpensive tool to improve treatment decisions by identifying patients who are less likely to benefit from endovascular treatment despite its reduced invasiveness. It may also facilitate a more personalized and cost-effective treatment approach for patients with AAA. Our group plans future research efforts to further investigate the utility of incorporating body composition into a comprehensive risk stratification model before aortic surgery.

## Figures and Tables

**Table 1:** Demographic characteristics of the study population with comparison between EVAR and OSR patients.

	All (*n* = 477)	OSR (*n* = 250) (52.4%)	EVAR (*n* = 227) (47.6%)	*p*-Value
Age, years	73.4 ± 8.0	70.8 ± 7.6	76.3 ± 7.5	<0.001 *
Male, *n* (%)	431 (90.4)	228 (53)	203 (47)	0.308
Weight, Kg	78.2 ± 14.6	78.2 ± 13.9	78.3 ± 15.5	0.380
Body mass index (Kg/m^2^)	26.4 ± 4.6	26.5 ± 4.7	26.3 ± 4.5	0.505
Hospital stay, *n*	9.9 ± 9.5	12.0 ± 9.4	7.7 ± 9.0	0.012 *
Hospital stay after intervention, *n*	8.4 ± 8.9	10.6 ± 8.9	6.2 ± 8.3	<0.001 *
Coronary diseases, *n* (%)	239 (50)	116 (46)	123 (54)	0.089
Dyslipidemia, *n* (%)	288 (60.4)	152 (60.4)	136 (60)	0.843
Hypertension, *n* (%)	375 (78.6)	196/250	180/227	0.730
Neurological diseases (dementia), *n* (%)	13 (2.7)	6 (2.4)	7 (3)	0.228
Smoking, *n* (%)	221 (46.3)	120 (48)	101 (44)	0.443
Peripheral artery disease, *n* (%)	96 (20)	46 (18)	50 (22)	0.312
Cardiac failure, *n* (%)	11 (2.3)	4 (4.4)	7 (3.1)	0.281
Cardiac valvular disease, *n* (%)	35 (7.3)	19 (7.6)	16 (7)	0.818
COPD, *n* (%)	103 (21.6)	50 (20)	53 (23.3)	0.437
Anticoagulant therapy, *n* (%)	73 (15.3)	26 (10.4)	47 (20.7)	0.002 *
Chronic renal failure, *n* (%)	91 (19.1)	36 (14.4)	55 (24.2)	0.006 *
Diabetes, *n* (%)	75 (15.7)	34 (13.6)	41 (18)	0.107
Stroke, *n* (%)	51 (10.7)	26 (10.4)	25 (11)	0.829
Cancer, *n* (%)	91 (19.3)	41 (16.4)	51 (22.4)	0.159
Obesity, *n* (%)	44 (9.2)	20 (8)	24 (10.5)	0.345
Clinical presentation asymtomatic, *n* (%)	407 (85.3)	202 (80.8)	205 (90.3)	0.001 *
Clinical presentation symtomatic, *n* (%)	70 (14.7)	48 (19.2)	22 (9.7)	0.001 *
Diameter, *n* (%)	57.2 ± 15.2	55.5 ± 12.8	58.7 ± 17.1	0.034 *
Necessity of blood transfusions, *n* (%)	69 (14.5)	58 (23.2)	11 (4.8)	0.001 *
Creatinin (mg/dL)	458 ± 82	104.3 ± 79.4	105.0 ± 85.0	0.907
SMA, cm^2^	146.6 ± 33.5	149.5 ± 35.1	143.5 ± 31.2	0.085
SMI, cm^2^/m^2^	49.5 ± 10.8	50.3 ± 11.2	48.7 ± 10.3	0.173
VAT, cm^2^	212.1 ± 194.2	194.4 ± 104.0	231.7 ± 259.2	0.064
SAT, cm^2^	161.3 ± 59.7	161.7 ± 60.6	160.9 ± 58.9	0.907

Abbreviations: skeletal muscle area (SMA), skeletal muscle index (SMI), visceral adipose tissue (VAT), subcutaneous adipose tissue (SAT), chronic obstructive pulmonary disease (COPD), open surgical repair (OSR), and endovascular aneurysm repair (EVAR); * *p*-value < 0.05.

**Table 2:** Body composition parameters predictors of length of hospital stay after open aortic surgical repair and endovascular aortic repair.

				OSR						EVAR		
	Crude Model			Adjusted Model								
	Β-Coefficient	CI 95%	*p*-Value	Β-Coefficient	CI 95%	*p*-Value	Β-Coefficient	CI 95%	*p*-Value	Β-Coefficient	CI 95%	*p*-Value
SMA	−0.032	−0.050–0.032	0.665	0.001	−0.42–0.043	0.991	−0.200	−0.111; −0.017	<0.001	−0.194	−0.114; −0.010	0.019
Sarcopenic	0.046	−2.776–5.292	0.539	0.391	−3.240–4.841	0.696	−0.247	−2.853; −11.013	<0.001	−3.224	−2.734–11.370	0.002
SMI	−0.006	−0.137; 0.125	0.934	0.025	−0.113–0.157	0.747	−0.156	−0.305; −0.004	0.045	0.120	−0.289–0.034	0.120
VAT	0.020	−0.012–0.016	0.927	0.357	−0.012–0.018	0.722	−0.063	−0.008–0.003	0.409	0.231	−0.009–0.001	0.231
SAT	0.062	−0.014; 0.034	0.402	0.081	−0.011–0.037	0.290	0.219	−0.023–0.028	0.402	0.110	−0.024–0.027	0.912

Abbreviations: skeletal muscle area (SMA), skeletal muscle index (SMI), visceral adipose tissue (VAT), subcutaneous adipose tissue (SAT), open surgical repair (OSR), and endovascular aneurysm repair (EVAR); Crude and adjusted models for selected covariates to examine potential confounding effects are shown for both procedures.

**Table 3:** Body composition parameters predictors of 1-year mortality after OSR and EVAR.

	OSR			EVAR		
Biompedentiometry Parameters	HR	CI 95%	*p*-Value	HR	CI 95%	*p*-Value
SMA	0.9861	1.001–1.027	0.030 *	1.301	0.947–0.996	0.026 *
SMI	0.9567	0.988–1.105	0.117	1.066	0.863–1.020	0.134
VAT	0.9952	1.001–1.009	0.012 *	1.002	0.990–1.005	0.490
SAT	0.993	0.999–1.015	0.065	1.010	0.998–1.021	0.094

Abbreviations: skeletal muscle area (SMA), skeletal muscle index (SMI), visceral adipose tissue (VAT), subcutaneous adipose tissue (SAT), open surgical repair (OSR), and endovascular aneurysm repair (EVAR); Hazard Ratio (HR), * *p*-value < 0.05.

**Table 4:** Body composition parameters predictors of long-term mortality after Open Aortic Surgical Repair (OSR) and Endovascular Aortic Repair (EVAR).

	OSR						EVAR					
Biompedentiometry Parameters	Univariate		Multivariate									
	HR	CI 95%	*p*-Value	HR	CI 95%	*p*-Value	HR	CI 95%	*p*-Value	HR	CI 95%	*p*-Value
SMA	1.003	0.99–1.004	0.794	1.001	0.994–1.007	0.723	0.993	0.986–0.999	0.004 *	0.989	0.982–0.996	0.003 *
SMI	0.995	0.972–1.019	0.708	0.996	0.973–1.021	0.802	0.986	0.967–1.003	0.178	0.977	0.957–0.998	0.032 *
VAT	1.000	0.997–1.002	0.977	1.005	0.997–1.002	0.885	0.998	0.997–1.002	0.171	0.998	0.996–0.999	0.034 *
SAT	1.000	0.996–1.004	0.847	1.000	0.996–1.004	0.811	0.997	0.994–1.001	0.255	0.996	0.992–1.000	0.112

Abbreviations: skeletal muscle area (SMA), skeletal muscle index (SMI), visceral adipose tissue (VAT), subcutaneous adipose tissue (SAT), open surgical repair (OSR), and endovascular aneurysm repair (EVAR); Hazard Ratio (HR), * *p*-value < 0.05. Crude and adjusted models for selected covariates to examine potential confounding effects are shown for both procedures.

## Data Availability

The data presented in this study are available upon request to the corresponding author. The data are not publicly available due to participant confidentiality.

## Supplementary Materials

The following supporting information can be downloaded at: https://www.mdpi.com/article/10.3390/nu16183205/s1, Table S1. Body composition characteristics by gender. Table S2. Body composition parameters predictors of length of hospital stay after open aortic surgical repair and endovascular aortic repair by gender. Table S3. Demographic characteristics of the study population by sarcopenic and non-sarcopenic status. Table S4. Body composition parameters predictors of 3-year mortality after OSR and EVAR.

## Abbreviations

Abdominal aortic aneurysm (AAA); endovascular aneurysm repair (EVAR); open aortic surgery (OSR); skeletal muscle index (SMI); skeletal muscle area (SMA); visceral adipose tissue (VAT); subcutaneous adipose tissue (SAT); computed tomography (CT).
