# Supplementary material for: The Impact of Body Composition on Mortality and Hospital Length of Stay after Endovascular and Open Aortic Aneurysm Repair: A Retrospective Cohort Study
Source: Nutrients. 2024 Sep 22;16(18):3205. doi: 10.3390/nu16183205 (PMC11434744; doi:10.3390/nu16183205)
Supplement: Supplementary file 1 [file nutrients-16-03205-s001.zip › nutrients-3193240-supplementary.pdf]

Supplementary Table S1. Body composition characteristics by gender

	**Males**			**Females**		
	**OSR**	**EVAR**		**OSR**	**EVAR**	**p-value**
**SMA**	153.6±32.5	148.0±28	0.290	98.6±25.2	100.5±23.4	0.708
**SMI**	51.4±10.6	49.7±9.9	0.249	37.0±9.4	38.6±7.8	0.749
**VAT**	203.7±100.8	242.0±268.0	0.052	82.0±74	127.92	0.249
**SAT**	162.0±56.6	157.7±57.1	0.810	157.9±99.9	191.5±68.8	0.396

Abbreviations: skeletal muscle area (SMA), skeletal muscle index (SMI), visceral adipose tissue (VAT), subcutaneous adipose tissue (SAT), open surgical repair (OSR), and endovascular aneurysm repair (EVAR); *p-value<0.05.

Supplementray Table S2. Body composition parameters predictors of length of hospital stay after open aortic surgical repair and endovascular aortic repair by gender

	**All populations**					**OSR**			**EVAR**			
	B-coefficient	p-value	B-coefficient	p-value	B-coefficient	p-value	B-coefficient	p-value	B-coefficient	p-value	B-coefficient	p-value
	Males		Female	p-value	Male		Female		Male		Female	
SMA	-0.020	0.730	-0.250	0.223	-0.310	0.697	0.068	0.875	-0.370	0.673	-0.284	0.431
Sarcopenic	0.083	0.144	0.214	0.255	0.900	0.242	0.083	0.861	0.081	0.335	0.279	0.685
SMI	0.110	0.849	-0.230	0.196	-0.002	0.980	0.281	0.511	0.005	0.952	-0.404	0.262
VAT	-0.070	0.225	0.217	0.930	0.005	0.948	-0.008	0.994	-0.089	0.271	0.081	0.807
SAT	0.035	0.541	-0.078	0.715	0.095	0.227	0.154	0.736	-0.077	0.340	0.029	0.923

Abbreviations: skeletal muscle area (SMA), skeletal muscle index (SMI), visceral adipose tissue (VAT), subcutaneous adipose tissue (SAT), open surgical repair (OSR), endovascular aneurysm repair (EVAR); *p-value<0.05.

Supplementray Table S3. Demographic characteristics of the study population by sarcopenic and non-sarcopenic status.

	**Sarcopenic**	**Non-Sarcopenic**	**p-value**
EVAR, n(%)	27 (48.2%)	149 (47%)	ns
OSR, n(%)	29 (51.8%)	168 (53%)	ns
Age, years	77.2±7.9	72±7.8	<0.001
Body mass index (Kg/m2)	22.7±3.5	26.9±4.03	<0.001
Hospital stay, n	9.7±8.6	13.7±16.1	<0.001
Hospital stay post procedure, n	8.1±7.8	11.8±15.5	<0.001
Dyslipidemia, %	46%	64%	<0.001
Hypertension, %	78%	77%	ns
Diabetes, %	9	19	0.044
Smoking, %	46%	54%	ns
Creatinin,mg/dl	104±92	91 ±45	ns

Supplementray Table S4. Body composition parameters predictors of 3-year mortality after OSR and EVAR

	OSR			EVAR		
**Biompedentiometry parameters**	**HR**	**CI 95%**	**p-value**	**HR**	**CI 95%**	**p-value**
**SMA**	0.982	0.970-0.994	**0.040***	1.005	0.996-1.015	0.220
**SMI**	0.954	0.920-0.989	**0.01***	1.006	0.967-1.047	0.730
**VAT**	0.997	0.993-1.000	0.08	1.002	0.999-1.005	0.162
**SAT**	0.998	0.991-1.00	0.551	1.001	0.995-1.007	0.565
